# The Rating Form of IBD Patient Concerns: Translation, Validation, and First Implementation of the Greek Version

**DOI:** 10.1155/2017/6267175

**Published:** 2017-04-25

**Authors:** Konstantinos Argyriou, Eleftheria Roma, Andreas Kapsoritakis, Eirini Tsakiridou, Konstantinos Oikonomou, Anastassios Manolakis, Spyridon Potamianos

**Affiliations:** ^1^Department of Gastroenterology, University Hospital of Larissa, Mezourlo 1, 41110 Larissa, Greece; ^2^First Department of Paediatrics, Gastroenterology Unit, University of Athens, Thevon and Levadias, 11527 Athens, Greece

## Abstract

*Background*. The rating form of IBD patients' concerns (RFIPC) provides a unique assessment of the worries and concerns of inflammatory bowel disease (IBD) patients. Our aims were primarily to validate the Greek version of RFIPC and secondarily to describe the pattern of Greek patients'concerns. *Methods*. After translating RFIPC, the questionnaire was given to IBD patients at baseline and after 12 weeks. The questionnaire's measuring properties were evaluated based on the consensus-based standards for the selection of health status measurement instruments (COSMIN) recommendations. Premediated factorial structures were tested for goodness of fit with confirmatory factor analysis (CFA). *Results*. At baseline, 200 patients (94 with Crohn's disease) completed RFIPC. After 12 weeks, the first 100 patients recompleted the questionnaire. CFA results were consistent with a slightly modified than the original factorial structure. Cronbach's *α* and intraclass correlation coefficients were high. RFIPC scores negatively affected the quality of life. RFIPC was sensitive to detect important changes in patients' condition and was able to discriminate between remission and active disease. Disease activity, full time employment, celibacy, and low education were associated with higher scores. *Conclusion*. The Greek version of RFIPC is a reliable, valid, and responsive tool to assess Greek IBD patients' concerns.

## 1. Introduction

Inflammatory bowel disease (IBD), including ulcerative colitis (UC) and Crohn's disease (CD), is a chronic relapsing condition of the gastrointestinal tract. [1] Over their longitudinal course, IBD imposes changes in patients' everyday life that adversely affect their health-related quality of life (HRQoL) [2–4].

Living with IBD has been associated with an increased level of emotional distress, anxiety, and depression [5–7].

Psychological changes, apart from their negative impact on patient's HRQoL, increase the feeling of powerlessness that may reflect to the level of worries and concerns that normally follow chronic disease [8–11].

Patients' knowledge and beliefs about their illness, personality traits, illness management behavior, personal attitudes, and expectations may all affect disease-related concerns [11].

The identification of worries and concerns in IBD patients may reveal important issues for the patient, but not obvious to healthcare workers which are of particular importance when organizing interventions that aim to improve patients' HRQoL [12].

The rating form of IBD patients' concerns (RFIPC) is a unique psychometric instrument that provides a reliable and accurate description of the pattern of patients' IBD-related worries and concerns [12].

To date, RFIPC has been used in both longitudinal and cross-sectional studies, contributing to the understanding of the burden of IBD [13–15].

However, little is known regarding patients' worries and concerns in Greece, since RFIPC has not been officially translated in Greek language.

The aims of our study were primarily to translate and evaluate the measurement properties of the Greek version of RFIPC. Secondarily, we aimed to describe IBD-related worries and concerns in Greek patients as well as to assess whether they were associated with the sociodemographic and clinical characteristics of our population.

## 2. Materials and Methods

### 2.1. Patients and Design

The study took place at the University Hospital of Larissa which constitutes the sole tertiary referral center for IBD patients in Central Greece. The study was approved by the Ethics committee and the Advisory board of the University Hospital of Larissa (D.N.18484/17-04-2013). All data were handled anonymously. Participation was voluntary, and the patients could withdraw from the study at any time.

During a 2-year period (2014–2016), all consecutive patients came from our IBD outpatient clinic or from the gastroenterology department where they had been admitted for an IBD-related cause, screened for eligibility, and entered the study.

Inclusion criteria were the patients to be adults, to know fluent Greek reading and writing, and to have IBD per standard criteria [16, 17]. Patients diagnosed with a major neuropsychiatric disorder prior to the diagnosis of the intestinal disease or other comorbidities were excluded from the study, to avoid overestimation or underestimation in the assessment of disease-related worries.

The data were collected by the same investigator, who was not the treating doctor of the patient, during a personal interview. All interviews were held during the follow-up visit of the patients or within two days after their admission. Data collection included a diary card with the characteristics of the population, the Greek version of RFIPC, and two HRQoL measurement tools that were to be used for testing construct validity.

To test the responsiveness of the Greek version of RFIPC, the first 100 patients were asked to complete the questionnaire for the second time. Throughout literature, the time interval between the administrations has not been accurately predefined but it should have been long enough to prevent recall bias. For RFIPC, the time frame was ranging from 15 days to 6 months [18, 19]. Consequently, in our study, we set this frame approximately at the half of the above period, at twelve weeks (2nd visit). Patients' change in health status compared to initial recruitment (baseline–1st visit) was assessed by the participants themselves using a five-point Likert type scale and confirmed with the use of the appropriate per disease type clinical index. In this scale, the value 1 corresponded to the worst while the value 5 to the best status.

### 2.2. Population Characteristics

The sociodemographic characteristics collected were gender, age, place of residence, marital, and employment status of each patient.

IBD-related variables were smoking, disease type, disease duration, and disease activity. Activity was assessed using the clinical indices Harvey-Bradshaw index for CD and Simple Clinical Colitis Activity Index for UC [20, 21]. The activity data that were collected were referring to the patient's activity status at the time of recruitment (short activity) and the preceding 3 years (long activity). Long active disease was considered the disease for which activity had been recorded in most of the follow-up visits of the patient over the previous 3 years, according to the medical record of each patient. Regarding activity, IBD patients were divided into two categories. Cut-off was considered for all indices the value of 5. Values < 5 were considered to indicate clinical remission.

### 2.3. Questionnaires

#### 2.3.1. RFIPC

RFIPC consists of 25 item questions, each of which is scored on a horizontal visual analogue scale 0–100 mm. Extreme values 0 mm indicate no concern while 100 mm the greatest concern. The questions are of the following form “Because of your condition, how concerned are you with … ?”. In the original English version, exploratory factor analysis using maximum likelihood method with varimax rotation grouped 22 of the 25 items into four factors: disease impact, complications, sexual intimacy, and body stigma. Three items were excluded due to low factor loadings. The score for each question ranges from 0–100. The mean score of all 25 items yields the “sum score” [12].

#### 2.3.2. RFIPC Translation Process

The translation process of RFIPC was conducted in line with the guidelines of the Rome Foundation and held in three stages [22]. The Rome Foundation appointed a gastroenterologist with proven experience in IBD as supervisor and counselor of the whole process.

In the first stage, two professional Greek translators with experience in medical translations worked independently of each other and made the translation of the questionnaire from English to Greek. From their work, two versions of RFIPC in the target language emerged. The two translators together with the Rome Foundation-appointed clinician compared the two forward versions to identify differences and conduct a reconciliation process.

In the second stage, the common version, resulted from the previous stage, was translated back to English from a third professional translator who was a native English speaker and fluent in Greek.

The backward translation and the original English version, in the third stage, were compared question by question for similarity of language (literal translation) and comparability of interpretation (cultural adaptation). In the final stage, other than the supervising consultant, 3 gastroenterologists experienced in IBD patients and two Greek patients who spoke fluent English were involved. Patient participation was decided to ensure that the Greek version of RFIPC is in line with the mentality of the target population.

The final form of the Greek version of RFIPC was firstly approved by the supervising consultant and then followed its acceptance by the administrative board of Rome Foundation (see Supplementary Material available online at https://doi.org/10.1155/2017/6267175).

#### 2.3.3. HRQoL

HRQoL of IBD patients was assessed with the generic questionnaire Short Form-36 (SF-36) and the disease-specific Inflammatory Bowel Disease Questionnaire (IBDQ).

SF-36 includes 36 questions that are divided into 8 multi-item dimensions consisting of physical functioning, physical role, bodily pain, general health, vitality, social functioning, emotional role, and mental health [23].

IBDQ consists of 32 items that are divided into four dimensions, assessing bowel symptoms, systemic symptoms, emotional, and social function [24].

Both questionnaires had been translated and validated in Greek language in other studies and had been found accurate and reliable [25, 26].

#### 2.3.4. Statistical Analysis

Descriptive statistics was used to describe the study population.

The evaluation of the measurement properties of the questionnaire was conducted in accordance with international recommendations [27–29].

Confirmative factor analysis (CFA) was performed on the variance-covariance matrix of the RFIPC items to test the good fit of our data in premeditated structural models [12, 19, 30].

The fit of our data was assessed using chi-square test and the comparable fit index (CFI). Bad fit was evaluated by the root mean squared error of approximation (RMSEA). For RMSEA, cut-off value for ideal adjustment was 0.06. However, RMSEA values up to 0.8 were acceptable [31].

Internal consistency was tested by calculating Cronbach's alpha coefficient. Cronbach's alpha coefficient values > 0.7 indicate strong correlation.

Spearman correlation coefficients were used to assess the relationship among RFIPC and SF-36 and IBDQ scores.

To test the responsiveness of RFIPC, intraclass correlation coefficients (ICC) were used. An ICC > 0.7 was considered the minimum standard for reliability.

Wilcoxon signed-rank test and Cohen's *d* have been used to follow up RFIPC scores between the two visits. Values of 0.2, 0.5, and 0.8 were regarded as small, medium, and large effect sizes, respectively.

Measurement error was represented by the standard error of measurement [SEM = SD1 × √(1 − ICC), SD1: standard deviation at 1st visit]. The smallest detectable change (SDC) for the individual (SDC_IND_) and the group score (SDC_GROUP_) were calculated according to Terwee et al. [32].

The relationship among RFIPC scores and patient characteristics was evaluated by Mann Whitney Wilcoxon test for two groups and Kruskal-Wallis test when comparison involved more than two groups. Comparisons between continuous variables were performed with Spearman's correlation coefficients.

Factors significantly associated with RFIPC scores were entered in a linear regression analysis that was performed stepwise (backward elimination of variables).

The significance level was set at *p* = 0.05. For our analysis, we used the statistical software for windows, SPSS 17, and IBM AMOS 24.0.

## 3. Results

Two hundred patients out of 253 were eligible and entered the study. 86.5% came from our IBD outpatient clinic while the rest came from the gastroenterology department where they had been admitted for an IBD-related cause. Patients' characteristics are shown in Table 1.

### 3.1. Face Validity and Cross-Cultural Adaptation

Face validity and cross-cultural adaptation of RFIPC were assessed during the final stage of the translation process.

### 3.2. Description of Worries and Concerns of IBD Patients

Table 2 lists the mean scores of the 25 items of RFIPC from the most to least concern for the total population and per disease type.

The mean RFIPC sum score was 44 for the total population. The top five concerns were related to the unknown nature of the intestinal disease, the loss of self-control, the access to quality medical care, the side effects of treatment, and the energy level. In contrast, IBD patients were less concerned about their ability to have children. Per disease type, there were no significant differences in the type and level of concerns. Therefore, further analysis was performed on the total population.

### 3.3. Structural Validity

Basing on literature and our clinical observation, we tested our data for goodness of fit in 4 CFA models.

The first model was based on the assumption that all 25 items can be loaded into one single factor. This model was making a unidimensional assessment of patients' concerns and was the most widely used.

The second model was the one proposed by Drossman et al. which evaluated the level of concerns in 4 factors: disease impact, complications, sexual intimacy, and body stigma.

The third model arose from the translation of the questionnaire in the Norwegian language and partitioned patients' concerns in 6 factors: influence of disease, expectations, healing, intimacy, stigma, and complications.

The fourth and final model was the second model with the addition of correlated error terms between questions 16 and 17 that was based on clinical observation; ostomy for example is often a consequence of surgery in IBD patients.

**Figure 1:**
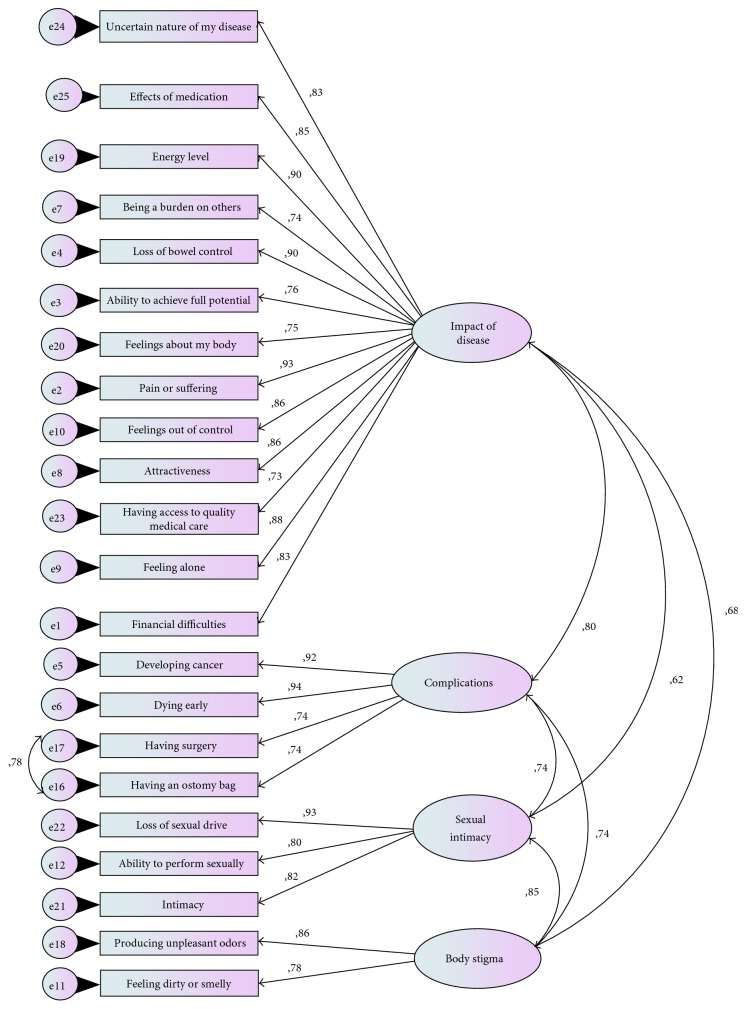
Illustrative representation of the adapted four-structural model of RFIPC (standardised factor loadings).

The results of CFA analysis are shown in Table 3. The first model showed poor fit. Better fit but still insufficient were found for the second and the third CFA models. Among the four CFA models, our data showed the most adequate fit to the fourth model. Chi-square test remained significant in all models. However, since it may remain significant even in models with excellent fit, the combined evaluation of the other indices reduced the chance of rejecting good fitting models. In Figure 1, it is shown in the structural model that our data had the most adequate fit.

Basing on the fourth structural model, the mean dimensional scores of RFIPC are shown in Table 4 for the total population and per disease type.

### 3.4. Internal Consistency

Cronbach coefficients (*α*) were calculated for each factor in the fourth model. For the 25 items, *α* was 0.95; for factor 1, disease impact, *α* was 0.96; for factor 2, complications, *α* was 0.92; for factor 3, sexual intimacy, *α* was 0.88; and for factor 4, body stigma, *α* was 0.78.

### 3.5. Floor-Ceiling Effect (Content Validity)

The proportion of patients who recorded the lower (<10) score in each of the four factors of the RFIPC was ranging from 0.5 to 7.5%. Similar was the proportion of patients that achieved the lower score in the 25 items of RFIPC with the exception of patients' concern about their ability to have children, where the lower score was recorded by 36.5% of participants. There was no missing data in our analysis. No ceiling effects were recorded.

### 3.6. Criterion Validity

Criterion validity was not tested since no other questionnaire or gold standard that measures IBD patient's worries and concerns exist.

### 3.7. Construct Validity

RFIPC subscores were significantly associated with the HRQoL scores irrespective of the measuring instrument used (*p* < 0.001). Moderate negative correlations were recorded between RFIPC subscores and the subscores in the SF-36 (*r* = 0.334–0.470, *p* < 0.001). In addition, moderate to high correlations were recorded between RFIPC subscores and IBDQ (*r* = 0.433–0.575, *p* < 0.001).

### 3.8. Discriminant Ability

Of the 200 patients enrolled in the study, 67 were in remission and 133 had active disease. Between the two groups, significant difference was found in all four RFIPC dimensions (*p* < 0.001).

### 3.9. Responsiveness

12 weeks after recruitment, 33 patients self-reported their condition to be unchanged. 67 patients reported improvement or deterioration. The results of test-retest reliability are shown in Table 5. ICC for absolute agreement in stable patients showed high reliability. As shown in Table 5, the questionnaire had good sensitivity to detect changes in patients' condition.

### 3.10. Measurement Error

SEM, SDC_IND_, and SDC_GROUP_ were calculated for each domain of RFIPC and for sum score. For disease impact, SEM, SDC_IND_, and SDC_GROUP_ were 1.9, 5.29, and 0.9, respectively; for complications, SEM, SDC_IND_, and SDC_GROUP_ were 6.26, 17.34, and 3.02, respectively; for sexual intimacy, SEM, SDC_IND_, and SDC_GROUP_ were 7.86, 21.78, and 3.79, respectively; for body stigma, SEM, SDC_IND_, and SDC_GROUP_ were 8.72, 24.17, and 4.21, respectively; and for sum score, SEM, SDC_IND_, and SDC_GROUP_ were 2.47, 6.85, and 1.19, respectively.

### 3.11. Relationships between RFIPC Subscores and Population Characteristics

Of all the sociodemographic characteristics examined, being single (celibacy) and full-time working status were associated with significantly higher scores in all areas of RFIPC (*p* < 0.001). Male sex was associated with higher scores on the complications domain (*p* = 0.038) whereas low education levels, in all domains of concerns (*p* = 0.049–0.001) except from sexual intimacy (*p* = 0.272).

Regarding disease-related characteristics, activity status (short and long) was associated with higher scores in all areas of RFIPC (*p* < 0.001). Smoking was related to higher level of concerns in all domains (*p* = 0.12–0.48) except from disease impact (*p* = 0.113) while disease duration in the domains of sexual intimacy and body stigma (*p* = 0.028–0.005).

In multivariate analysis, short activity (disease impact *b* = 0.132, *p* = 0.043; complications *b* = 0.251, *p* < 0.001; sexual intimacy *b* = 0.335, *p* < 0.001; and body stigma *b* = 0.352, *p* < 0.001) and full-time working were independently associated with higher level of concerns in all four domains (disease impact *b* = 0.293, *p* < 0.001; complications *b* = 0.217, *p* < 0.001; sexual intimacy *b* = 0.153, *p* = 0.021; and body stigma *b* = 0.214, *p* = 0.001). Of the remaining factors, celibacy was associated with higher concerns in all domains except from body stigma (disease impact *b* = 0.148, *p* = 0.016; complications *b* = 0.165, *p* = 0.009; and sexual intimacy *b* = 0.149, *p* = 0.027); long activity in the domains of disease impact (*b* = 0.285, *p* < 0.001) and complications (*b* = 0.229, *p* = 0.001); and low education with higher scores in the domain of disease impact (*b* = 0.162, *p* = 0.005).

## 4. Discussion

As chronic diseases, IBD causes uncertainty and imposes cancellations and restrictions on patients' everyday life that adversely affect their HRQoL [11, 33, 34].

HRQoL provides a comprehensive assessment of the patient's point of view and experience of the disease and is influenced by factors related not only with the disease and its treatment but also with the patients' personality [35].

Disease-related worries and concerns are an integral part of patients' personalities that are strongly related with their HRQoL. Their knowledge allows a better understanding of the dimensions of chronic disease, and their management has been shown to be of importance since worries and concerns can affect a person's ability to adapt to the disease as well as the subsequent compliance to treatment [11, 36, 37].

RFIPC allows an accurate and reliable assessment of worries and concerns of IBD patients [12].

To date, RFIPC has been translated into 7 languages. RFIPC has been used in studies to characterize HRQoL, to understand the type and the degree of worries and concerns in IBD patients, and to make comparisons between different populations and determine their actual needs [12, 18, 19, 38–41].

However, in Greece, little is known regarding patients' disease-related worries and concerns since RFIPC had not been officially translated and validated in Greek language.

The primary aim of our study was the translation and the evaluation of the measuring properties of the Greek version of RFIPC. Secondarily, we aimed to describe IBD-related worries and concerns in Greek patients as well as to assess whether they were associated with the sociodemographic and clinical characteristics of our population.

The translation of RFIPC was performed according to Rome Foundation criteria, and its validation was in line with COSMIN checklist [22, 27–29].

Over the cultural adaptation of RFIPC, in line with the different social, economic, and cultural conditions of each country, the original factorial structure suggested by the creators of the questionnaire showed differentiations to a lesser or greater extent [19, 30]. This finding was also observed in our study where we found that the factorial structure of RFIPC as proposed by Drossman et al. can be replicated with slight modification.

In agreement with studies that evaluated the RFIPC in other populations, the validity of the Greek version was found to be high [12, 18, 19, 30].

The questionnaire was able to distinguish between patients who were in remission and those who had active disease accurately.

RFIPC sensitivity to detect changes that were reported as having occurred in the patients' health condition was good with statistically significant differences to be recorded in all of its four domains. In contrast, as expected, no significant differences were identified in patients whose condition remained stable.

In our study, it was observed that the sum score of worries and concerns in IBD patients was higher than that observed during the translation process of RFIPC in Norway [19]. This finding is in accordance with the results of a previous cross-cultural study, confirming that patients with IBD from Southern Europe have higher level of concerns compared to those originating from the northern countries [41].

However, the unidimensional approach of patients' concerns as expressed with the report and the comparison of the sum scores does not provide specific information about differences that exist concerning the type and the degree of concerns among different populations [19, 30]. This information can be provided if analysis would be performed item per item or in domains, providing additional details in the context of patients' concerns not only for cross-cultural comparisons but also for health care providers to identify their patients' special needs.

In our study, IBD patients were more concerned about the unknown nature of the intestinal disease, the loss of self-control, the access to quality medical care, the side effects of treatment, and their level of energy. Compared to other populations, our patients were to be more concerned about issues related to quality of care, self-control, and treatment [12, 14, 15, 19, 41]. These findings are possibly attributed to incomplete information of our patients on issues related to their health, the latest developments, and the side effects of current therapies. According to a recent study from Greece, approximately 50% of patients with IBD have incomplete information on the disease while only 31% are informed about the latest developments [42]. A group-based psychoeducational program could reduce this kind of uncertainty that follows chronic disease and has been put forward in order to improve patients' HRQoL.

Our patients have been found to be less concerned about their ability to have children. This finding is in accordance with other studies [12, 19]. However, the presence of that question in the scale of concerns has been criticized as it is associated with younger age and not for the entire range of the age of IBD patients [19].

Of the population characteristics, short activity and full-time work were independently associated with higher concerns in all four domains. Among the other factors, celibacy was associated with higher concerns in all domains except from body stigma, long activity with higher concern in the domains of disease impact and complications, and lower education with higher concerns in the domain of disease impact.

With the exception of symptomatic disease, there is no consensus as to the relationship of the other characteristics of the population to the level and type of concerns [12, 14, 15, 19]. However, the determination of the characteristics of the population in every society that are associated with higher level of concerns is important since it indicates subgroups of the population in which health care providers should show greater attention in order to maximize the therapeutic outcome.

One possible limitation of our study is that we only used clinical indices for the assessment of patients' activity status. The noninvasive nature of our study with the repetitive follow-up of our patients precluded the use of the more invasive indices. As there is no evidence to date that a specific marker will accurately represent the whole IBD patient population, the combination of less and more invasive markers may be more clinically suitable when attempting to evaluate disease activity. However, in clinical practice, symptom-based indices have been found to be reliable in detecting clinical meaningful changes in patients' condition with most treatment algorithms to be symptom-based at a point in time [43].

## 5. Conclusions

The Greek version of RFIPC allows the accurate and reliable determination of the level of concern of Greek IBD patients and can be used in future studies. Worries and concerns related to the unknown nature of the disease, the loss of self-control, the access to quality health care, the side effects of treatment, and the energy level predominated in our population. Other than active disease, lower education, celibacy, and full-time employment have been found to be related with higher level of concerns in our population suggesting possible target groups for future interventions.

## Supplementary Material

Supplementary Material. The approval of the translation by the Rome Foundation.

## Figures and Tables

**Table 1:** Patients' characteristics.

	CD^∗^ (*N* = 96)	UC^∗∗^ (*N* = 104)	*p* value
*Sex*			
Females	50 (52.1)	53 (51)	N/S^∗∗∗^
Age (mean)	39.2	42.1	N/S
*Residence*			
Rural	48 (50)	53 (51)	N/S
Urban	48 (50)	51 (49.0)	
*Level of education*			
<12 years	64 (66.7)	62 (59.6)	N/S
>12 years	32 (33.3)	42 (40.4)	
*Marital status*			
Single	25 (26.1)	40 (38.5)	0.01
Married	63 (65.6)	50 (48.1)	
Divorced/widowed	8 (08.3)	14 (13.4)	
*Employment status*			
Unemployed	20 (20.8)	20 (19.2)	N/S
Part-time employed	21 (21,9)	22 (21.2)	
Full-time employed	47 (49.0)	50 (48.1)	
Pensioner	8 (08.3)	12 (11.5)	
*Smoking*			
Yes	34 (35.4)	27 (26)	N/S
No	62 (64.6)	77 (74)	
*Duration (mean)*	9.11	9.47	N/S
*Short disease activity*			
Recession	30 (31.3)	37 (35.6)	N/S
Active	66 (68.7)	67 (64.4)	
*Long disease activity*			
No	68 (70.8)	74 (71.2)	N/S
Yes	28 (29.2)	30 (28.8)	

^∗^CD: Crohn's disease; ^∗∗^UC: ulcerative colitis; ^∗∗∗^N/S: nonsignificant; *p* < 0.05: statistically significant.

**Table 2:** Description of worries and concerns of the study population (mean scores and numerical rank).

	IBD^∗^ Mean (sd)	CD^∗∗^ Mean (rank)	UC^∗∗∗^ Mean (rank)
Unknown nature of disease	56,77 (10.46)	56.81 (1)	56.73 (1)
Feeling out of control	55,71 (8.94)	55.64 (2)	55.78 (2)
Having access to quality medical care	53,38 (7.03)	53.42 (3)	53.36 (3)
Fear of side effects	51,10 (9.72)	50.78 (4)	51.40 (4)
Energy level	49,91 (10.37)	49.71 (5)	50.10 (5)
Pain and suffering	49,66 (10.54)	49.24 (7)	50.05 (6)
Loss of bowel control	49,05 (10.28)	49.68 (6)	48.48 (7)
Having an ostomy bag	47,49 (17.88)	47.52 (8)	47.46 (8)
Attractiveness	46,49 (8.58)	46.42 (10)	46.57 (9)
Ability to achieve full potential	46,08 (6.95)	46.45 (9)	45.74 (11)
Having surgery	45,30 (15.80)	45.45 (11)	45.16 (13)
Dying early	45,19 (16.30)	44.46 (12)	45.87 (10)
Developing cancer	44,67 (14.01)	43.66 (16)	45.61 (12)
Feeling alone	44,19 (8.26)	44.28 (13)	44.12 (15)
Loss of sexual drive	44,06 (19.45)	43.35 (18)	44.71 (14)
Intimacy	44,01 (16.83)	44.20 (14)	43.85 (16)
Feelings about my body	43,42 (11.61)	43.82 (15)	43.06 (19)
Being a burden on others	43,39 (6.65)	43.25 (20)	43.53 (17)
Financial difficulties	43,23 (14.25)	43.28 (19)	43.18 (18)
Being treated as different	43,10 (6.73)	43.44 (17)	42.79 (20)
Ability to perform sexually	40,91 (16.65)	41.44 (21)	40.42 (21)
Feeling dirty	37,62 (20.40)	38.24 (22)	37.06 (22)
Produce unpleasant smell	37,11 (14.51)	37.45 (23)	36.79 (23)
Passing the disease on to your children	27,99 (7.87)	27.42 (24)	28.52 (24)
Ability to have children	15,05 (12.88)	15.20 (25)	14.92 (25)

^∗^IBD: inflammatory bowel disease; ^∗∗^CD: Crohn's disease; ^∗∗∗^UC: ulcerative colitis; sd: standard deviation.

**Table 3:** Confirmatory factor analysis: fit indices for the premediated structural models of RFIPC.

Model	Chi-square	df	CFI	RMSEA
1-factor	1564.8	275	0.74	0.154
4-factor	620.1	203	0.90	0.101
6-factor	533.8	194	0.91	0.094
Adapted 4-factor	455.3	202	0.94	0.079

1-factor model—single factor loading of the 25 items. 4-factor model—22 items loaded in 4 factors: disease impact, complications, sexual intimacy, and body stigma. 6-factor model—22 items loaded in 6 factors: impact of disease, expectancy, treatment, intimacy, stigma, and complications. Adapted 4-factor—22 items loaded in 4 factors permitting the correlated error terms between items 15 and 16: disease impact, complications, sexual intimacy, and body stigma.

**Table 4:** Mean dimensional scores of RFIPC.

	IBD^∗^ Mean (sd)	CD^∗∗^ Mean (sd)	UC^∗∗∗^ Mean (sd)
Disease impact	48.65 (08.12)	48.67 (08.27)	48.62 (08.02)
Complications	45.66 (14.39)	45.27 (13.96)	46.02 (14.83)
Sexual intimacy	42.99 (15.91)	42.99 (16.31)	42.99 (15.60)
Body stigma	37.37 (16.01)	37.84 (16.39)	36.93 (15.72)

^∗^IBD: inflammatory bowel disease; ^∗∗^CD: Crohn's disease ^∗∗∗^; UC: ulcerative colitis; sd: standard deviation.

**Table 5:** Responsiveness of RFIPC: comparison of scores between the 2 visits for the first 100 IBD patients. Intraclass correlation coefficients (ICC) calculated in those patients reported their condition to be unchanged (stable) in the second visit after 12 weeks. Effect-size calculated with Cohen's *d* in those reported changes in their condition.

	*n*	1st visit	2nd visit	Mean difference	ICC	Cohen's *d*	*p* value
*Stable patients*	33						
Disease impact	33	43.75	42.94	0.81	0.93		
Complications	33	38.76	36.40	2.36	0.87		
Sexual intimacy	33	32.71	36.26	−3.55	0.82		
Body stigma	33	28.42	30.53	−2.11	0.77		
*Improved patients*	44						
Disease impact	44	51.06	46.33	4.73		0.72	<0.001
Complications	44	52.02	45.07	6.95		0.71	<0.001
Sexual intimacy	44	48.01	41.68	6.33		0.52	<0.001
Body stigma	44	43.00	36.81	6.19		0.48	<0.001
*Deteriorated patients*	23						
Disease impact	23	49.49	53.73	−4.24		0.66	<0.001
Complications	23	50.20	58.48	−8.28		0.74	<0.001
Sexual intimacy	23	46.78	53.72	−6.94		0.55	<0.001
Body stigma	23	43.37	50.50	−7.13		0.55	<0.001
