# Supplementary material for: The Rating Form of IBD Patient Concerns: Translation, Validation, and First Implementation of the Greek Version
Source: Gastroenterol Res Pract. 2017 Apr 25;2017:6267175. doi: 10.1155/2017/6267175 (PMC5424483; doi:10.1155/2017/6267175)
Supplement: Supplementary file 1 — Supplementary Material. The approval of the translation by the Rome Foundation. [file 6267175.f1.pdf]

ROME FOUNDATION

To improve the lives of people with Functional GI Disorders

BOARD OF DIRECTORS
Douglas A. Drossman, MD
President
Chapel Hill, NC USA

Fernando Azpiroz, MD, PhD
Barcelona, Spain

Giovanni Barbara, MD
Bologna, Italy

Lin Chang, MD
Los Angeles, CA USA

William Chey, MD
Ann Arbor, MI, USA

John Kellow, MD, FRACP
New South Wales, Australia

Jan Tack, MD, PhD
Leuven, Belgium

Max Schmulson, MD
Mexico City, Mexico

Magnus Simrén, MD, PhD
Gothenburg, Sweden

Ami Sperber, MD MSPH
Beer-Sheva, Israel

William E. Whitehead, PhD
Chapel Hill, NC USA

Former Board Members
Robin C. Spiller, MD, MSC
Nottingham, England
2000-2014

Nicholas J. Talley, MD, PhD
Callaghan, Australia
1990-2011

Enrico Corazziari, MD
Rome, Italy
1989-2010

W. Grant Thompson, MD
Ottawa, Canada
1989-2008

Michel Delvaux, MD, PhD
Nancy, France
1999-2006

Executive Director
Wink Hilliard
Raleigh, NC 27628
Phone: 919-787-5859
Fax: 919-900-7646
Cell: 919-345-3927
wnhilliard@aol.com
whilliard@theromefoundation.org

Public Relations Director
Ceciel Rooker
Chapel Hill, NC USA
Phone: (843) 364-4292
crooker@theromefoundation.org

Administrators
Michele Pickard
Franklinton, NC USA
Phone: (919) 539-3051
mpickard@theromefoundation.org

Claudia Rojas
Chapel Hill, NC USA
crojas@theromefoundation.org

April 14, 2015

Eleftheria Roma MD
Emeritus Professor of Pediatric Gastroenterology
University of Athens, Greece

Re: Rome Foundation Approval of Translation to Greek

Dear Eleftheria,

We have received your documentation relating to the translation of the Rating Form of IBD Patient Concerns (RFIPC) into Greek.

Thanks to you and Dr. Konstantinos Argyriou for this excellent job and important contribution to the body of translated Rome Foundation material.

We are pleased to extend official Rome Foundation approval of the translation.

Sincerely,

Ami D. Sperber, MD, MSPH
Head, Rome Translation Project

CC: Dr. Konstantinos Argyriou

P.O. Box 6524; Raleigh, NC 27628
Phone: 919-787-5859 ~ Fax: 919-900-7646
www.theromefoundation.org
